# Incomplete Immune Recovery in HIV Infection: Mechanisms, Relevance for Clinical Care, and Possible Solutions

**DOI:** 10.1155/2012/670957

**Published:** 2012-03-14

**Authors:** Julie C. Gaardbo, Hans J. Hartling, Jan Gerstoft, Susanne D. Nielsen

**Affiliations:** Department of Infectious Diseases, Rigshospitalet, University Hospital of Copenhagen, 2100 Copenhagen, Denmark

## Abstract

Treatment of HIV-infected patients with highly active antiretroviral therapy (HAART) usually results in diminished viral replication, increasing CD4+ cell counts, a reversal of most immunological disturbances, and a reduction in risk of morbidity and mortality. However, approximately 20% of all HIV-infected patients do not achieve optimal immune reconstitution despite suppression of viral replication. These patients are referred to as immunological nonresponders (INRs). INRs present with severely altered immunological functions, including malfunction and diminished production of cells within lymphopoetic tissue, perturbed frequencies of immune regulators such as regulatory T cells and Th17 cells, and increased immune activation, immunosenescence, and apoptosis. Importantly, INRs have an increased risk of morbidity and mortality compared to HIV-infected patients with an optimal immune reconstitution. Additional treatment to HAART that may improve immune reconstitution has been investigated, but results thus far have proved disappointing. The reason for immunological nonresponse is incompletely understood. This paper summarizes the known and unknown factors regarding the incomplete immune reconstitution in HIV infection, including mechanisms, relevance for clinical care, and possible solutions.

## 1. Introduction

Treatment of HIV infection with highly active antiretroviral therapy (HAART) usually results in diminished viral replication and increasing CD4+ cell counts. When HAART is initiated, a biphasic response occurs with an initial high increase in CD4+ cells primarily due to reduced apoptosis and redistribution of memory CD4+ cells from lymphoid tissue, followed by a slower on-going increase in part generated from production of naïve CD4+ cells [1, 2]. For how long this increase proceeds is debatable, but cohort studies suggest CD4+ cell recovery for at least 5 years of HAART as long as the CD4+ cell count is <500 cells/*μ*L [3]. However, approximately 20% of all HIV-infected individuals fail to restore their CD4+ cell counts despite optimal treatment and fully suppressed viral replication [2, 4, 5]. These individuals are referred to as immunological nonresponders (INRs).

The definition of INR suffers from lack of consensus impeding the comparison of findings. Most often INRs are defined as having CD4+ cell counts <200 cells/*μ*L while the treatment duration needed for categorizing patients as INR is variable. Furthermore, some study groups define INR by the CD4+ cell increase in percentages, most commonly <20% increase from baseline [6–9]. On the other hand, there seems to be agreement that an adequate immune response to HAART should include a CD4+ cells count >500 cells/*μ*L, mainly because HIV-infected patients with this level of immune restoration have a morbidity and mortality rate approaching or comparable to those of HIV negative individuals [10]. Patients with CD4+ cell counts <500 cells/*μ*L are consequently classified as inadequate responders. Inadequate responders are a heterogeneous population since INR is included within this group of patients. Thus, a large group of inadequate responders, those with intermediate response with CD4+ cells counts between 200 and 500 cells/*μ*L, are poorly described, although they may have a morbidity and mortality rate distinct from INR as well as from those with adequate immune response [11]. In contrast, the increased risk of long-term morbidity and mortality in INR is widely accepted [11–13]. This demonstrates an obvious reason for delineating the cause of the poor immunological response seen in INR. It also emphasizes the need for additional treatment strategies for HAART. The scope of this paper is to focus on the immunological explanations for immunological nonresponse in patients with full virological responses, clinical relevance, and feasible solutions.

## 2. Explanations of Immunological Nonresponse

INR has been associated with a number of factors. Thus, it has been shown that older age, a long duration of the HIV infection prior to HAART, coinfection with hepatitis C, and a low CD4 nadir predispose to immunological nonresponse [13–17]. The CD4 nadir specifically appears to be critical for the recovery of CD4+ cells [14, 15]. However, none of these factors provide a full explanation for the lack of immune reconstitution in INR. As a result of this, immunological explanations have been proposed.

The CD4+ cell count in a given patient at any time is the result of production, destruction, and traffic between blood and lymphatic tissue. Thus, if the destruction exceeds the production, the CD4+ cell count decreases. INR may have alterations in the production of CD4+ cells resulting in a reduction of output as well as a destruction of CD4+ cells resulting in an increased turnover. Finally, the distribution of cells between blood and lymphatic tissue may be different in INR.

## 3. Production of CD4+ Cells

### 3.1. Thymus and Naive Cells

CD4+ cells are created from already existing CD4+ cells by proliferation, or they are produced in the thymus. The genesis of the T-cell receptor (TCR) takes place in the thymus only, and CD4+ cells generated in the thymus lead to immune reconstitution with a pool of CD4+ cells with full immunologic repertoire [1, 18]. Furthermore, HIV-infected patients with a large thymus have a better immune reconstitution and a broader immunological repertoire than patients with a small thymus [19, 20]. The thymus was thought to be only active in childhood; however it is replaced by fatty tissue with increase in age. It is now evident that the thymus can also be active in adulthood, particularly during circumstances with lymphopenia, as is the case with HIV infection [21, 22]. Thymic tissue has been visualized on computed tomography (CT) scans in HIV-infected adults, and the size of the thymus has been shown to be positively associated with naive CD4+ cell counts and total CD4+ cell counts [19, 23, 24]. However, the performance of CT scans is neither practical nor economically responsible in HIV-infected patients, and often thymic function is assessed indirectly as T-cell receptor excision circles (TRECs), as recent thymic emigrants (RTEs), or simply as the naive CD4+ cell count. TRECs are stable circular DNA fragments that are excised during the formation of TCR in the maturing T cell in the thymus, and TRECs are not replicated during cell division. Thus, the more immature CD4+ cells, the higher the TREC content. A large thymus on CT scans has been associated with a higher CD4+ TREC frequency in HIV-infected patients [19]. During the maturation process, T cells emigrate from the thymus into the periphery now classified as RTE [25], and after further maturation RTEs are classified as naive T cells. Thus, TRECS, RTEs and naive cells are all reasonable indirect measurements of thymic output, although the number of naive cells may be the result of thymic output as well as peripheral proliferation.

Thymic output is dramatically reduced with age, and the naïve cells are increasingly generated from proliferation (reviewed in [26]). RTEs express the surface marker platelet endothelial cell adhesion molecule-1 (PECAM-1) also known as CD31 [27, 28]. Proliferation leads to loss of CD31 and a lower TREC count, and therefore naïve cells in older individuals have decreasing proportions of CD31 and lower TREC counts [27, 28]. HIV leads to a disruption in the number and function of naïve CD4+ cells in blood as well as in lymphoid tissue [5, 29, 30]. After initiation of HAART, thymic output and the total numbers of naïve cells increase to subnormal levels, while the naïve T-cell proliferation decreases [5, 29–31]. Even 7 years of HAART rarely normalizes the naïve CD4+ cell counts to preinfection levels [32]. The naïve compartment in INR seems to be even more perturbed than in HIV-infected patients with a better immune reconstitution. Thus, one study found no residual thymic tissue on CT and PET scans in INR [33]. The same study found low, though detectable, thymopoiesis assessed as circulating RTE. In support of this, other studies have found decreased levels of naïve T cells and T cells expressing CD28 in INR [7, 34], suggesting an altered thymopoiesis in INR. The assumption that peripheral proliferation is a compensatory mechanism to altered thymopoesis in INR is supported by the finding of increased proportions of the peripheral proliferation marker Ki67 in INR, although the same study found similar levels of RTE in INR versus normal responders [35]. Similar levels of TREC in INR and normal responders have also been reported [36]. It is not known if a small thymus is predictive of INR. However, in a prospective study of 30 HIV-infected individuals, thymic CT scans were obtained to investigate the role of the thymus in cellular restoration after initiation of HAART. Individuals with abundant thymic tissue had higher naive CD4+ cell counts at weeks 2–24 than individuals with minimal thymic tissue [24]. Likewise, a large thymus has been shown to be associated with better immune reconstitution in other studies [23, 37].

**Figure 1:**
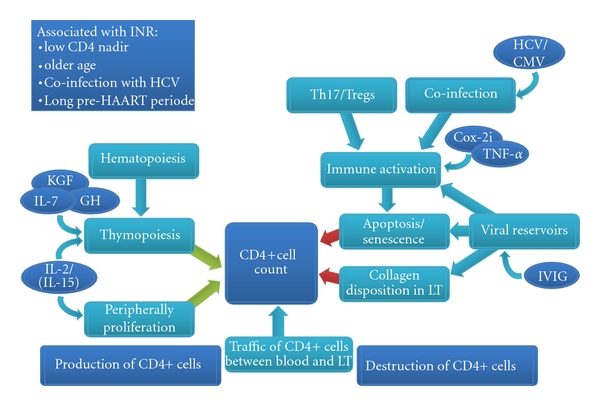
Factors influencing CD4+ cell count (LT: lymphatic tissue).

Another way to assess factors influencing the capability to produce CD4+ cells is to examine the response to treatment interruption. Thus, it has been demonstrated that a small thymic volume and a low level of memory CD4+ cells predict a faster loss of CD4+ cells during treatment interruption [38, 39]. This supports thymopoiesis as being essential in immune reconstitution. Interestingly, a newly published prospective study of thymectomized children showed that thymic tissue could be identified on magnetic resonance imaging scans in the majority of these children later on in life [40]. This highlights the great plasticity of the normal immune system, and together with the aforementioned findings it substantiates the evidence for permanent damage on the thymic tissue in INR (Figure 1).

### 3.2. Bone Marrow and Progenitor Cells

T cells mature in the thymus, but they originate from hematopoietic progenitor cells (HPCs) in the bone marrow (BM). Thus, a functional BM is crucial for thymopoiesis and possibly for immune reconstitution. Recently, HPC has been given much attention in the hopes of realizing a functional cure for HIV infection, more relevant than ever after the newly published report of eradication of HIV by transplantation of CCR5-deficient HPC in the so-called Berlin-patient [41]. HIV influences BM and HPC. First of all, impaired hematopoiesis in HIV infection has been shown in a number of studies [42–44]. Secondly, several studies have shown that a proportion of HPCs express the HIV receptors CD4, CXCR4, and CCR5 making them potentially susceptible to HIV infection (reviewed in [45]). Recently, infection of HPC with HIV was suggested [46], although it has been difficult to determine if these HPCs are actually infected according to the complexities of purifying and maintaining HPC in culture. Also, measurements of infection may be confounded by contamination with other cell types or maturation of HPC to monocytes during in vitro culture. Furthermore, like T cells, natural killer cells and B cells, including naïve B cells, seem to be depleted during HIV infection [47]. HIV-associated lymphopenia may therefore be explained by more upstream elements of lymphocyte development than reduced thymic output. In a study of BM from 12 INR compared to normal responders, an altered cytokine production was found, and a reduced growth of in vitro colonies was shown [34], suggesting impaired hematopoiesis as a contributing factor to poor immune reconstitution. However, studying the HPC is limited by the poor access to BM, and since HPCs enter the circulation [48], most studies are conducted on circulating HPC in the peripheral blood. Circulating HPCs have been found to decrease with disease progression and to be associated with CD4+ cell count [49], supporting the idea of BM and HPC as being essential in immunological reconstitution. This is further supported by in vitro studies, which has shown that treatment with the hematopoietic growth factor granulocyte-colony-stimulating factor (G-CSF) causes an increase in the numbers of circulating CD4+ cells [50]. Others have found that peripheral mononuclear blood cells (PBMCs) from HIV-infected patients placed on fetal thymus lobes from mice produced fewer CD4+ and CD8+ cells compared with PBMCs from uninfected controls. Also, fewer functional precursors in the HIV-infected patients were found [51]. This is consistent with a loss in the capacity of HIV-infected patients to produce functional T-cell progenitors in their peripheral blood. Thus, in theory dysfunctional BM and HPC might contribute to immunological nonresponse. However, so far very few studies have validated the number or function of HPC in patients with poor immune reconstitution.

### 3.3. Cytokines

Interleukin 7 (IL-7) is crucial in the T-cell homeostasis, and the IL-7 responsiveness is determined largely by the presence or absence of the IL-7 receptor (IL-7R) which is present on most mature T cells [52]. Furthermore, IL-7 is a modulator of peripheral T-cell homeostasis involved in maintaining the naïve T-cell pool by promoting their survival and inducing proliferation without switching naïve phenotype [53]. A negative correlation between IL-7 and CD4+ cell count is described. Consequently, HIV-infected patients show high levels of IL-7 and reduced levels of IL-7R compared to healthy controls [54, 55], consistent with the need for increased production of CD4+ cells and a downregulation of the receptor due to high plasma levels. A study found that a reduction of naive CD4+ cells in INR was associated with a reduced expression of IL-7R and in increased serum levels of IL-7 [6]. In addition, a higher stromal production of IL-7 in INR compared to normal responders has been observed [34, 56]. Considering IL-7 as an inducer of CD4+ cell production, these findings are not surprising in patients with a low CD4+ cell count. However, the interesting conclusion in relation to INR might be that the source of the CD4+ cells is impaired in INR, not the signals.

Like IL-7, interleukin 2 (IL-2) and interleukin 15 (IL-15), which are part of the gamma-chain cytokine family, are central regulators of T-cell proliferation, activation, and differentiation as well (reviewed in [57]). In contrast to IL-7, the production of IL-2 and IL-15 is compromised in HIV-infected patients (reviewed in [58] and [59]). Moreover, the production of IL-2 in blood from INR stimulated with phytohaemagglutinin has been shown to be decreased compared to HIV-infected patients with higher CD4+ cell counts [7]. Thus, in theory improving the regulation of IL-2 and IL-15 in HIV-infected patients might be beneficial as discussed later.

## 4. Destruction of CD4+ Cells

### 4.1. Immune Activation

Immune activation (IA) in the natural history of HIV infection covers a broad spectrum of cellular processes. Untreated HIV-infected patients display elevated markers of activation in most cell compartments, especially expression of the surface markers CD38 and HLA-DR on T cells [60–64]. Also, high levels of proinflammatory cytokines such as tumor necrosis factor alpha (TNFa), interleukin 6 (IL-6), and interleukin 1b (IL-1b) have been shown in plasma as well as in lymph nodes [65–68]. IA usually reflects a normal and healthy response upon infection with any pathogen, including HIV, as an effort to evade infection. However, it is well established that IA is linked to and predictive of disease progression, and IA has an additive or stronger prognostic value than does CD4+ cell count or viral load alone [60–64, 69–71]. This is highlighted by the fact that a rare subgroup of HIV-infected patients, elite controllers, who do not progress, and sustain normal CD4+ cell counts and undetectable viral loads despite lack of treatment have a lower IA than normal progressors do [72]. Also, the natural hosts of simian immunodeficiency virus (SIV), sooty mangabeys and African green monkeys, do not show any signs of increased IA, T cell turnover, or disease progression [73, 74]. Thus, IA is a key feature in HIV infection and disease progression, elegantly supported by the fact that rats develop pneumocystic pneumonia solely as a consequence of IA [75]. 

The reason for the strong predictive value of IA in HIV infection is uncertain. In the setting of untreated HIV infection, the level of IA might be determinant for how fast the turnover of T cells is, thereby being related to exhaustion. Indeed, it has been proposed that IA leads to CD4+ cell depletion because it erodes the naïve T-cell pool [76]. However, untreated and treated HIV infection are two different settings. IA declines when HAART is initiated, although like most other immunological parameters, it is not normalized [77–79]. IA is one of the best valued immunological features in INR, and a number of studies have shown elevated IA in INR compared to HIV-infected patients with a better immunological recovery [6, 7, 35, 80]. Assuming INR to have a dysfunctional immune system, the high level of IA could be a consequence of rather than a reason for poor immune reconstitution. Lack of association between the extent of CD4+ cell recovery and activation of CD8+ cells beyond the first year of successful HAART [81] as well as the opposite has been found [78]. Either way, it does not answer the question whether the increased IA is the result of more upstream deficits. Finally, increased IA during primary HIV infection has been proposed to be predictive of CD4+ cell depletion and poor response to HAART [82], suggesting that preinfection host factors may predict poor immune reconstitution.

Another aspect is the findings of an overweight of residual viremia detected by ultrasensitive assays in INR, which seems to be linked to IA [83]. This might reflect release of archived viruses from cellular reservoirs and might be a contributing factor to the higher levels of IA found in INR. Also, one study reports a higher frequency of CXCR4 virus in INR. They suggest X4 virus as players in the depletion of naïve T cells in INR by triggering persistent IA and bystander apoptosis via gp120-CXCR4 interactions [84], suggesting the coreceptor dominance to be involved in the level of immune reconstitution. Moreover, it is acknowledged that CCR5 virus dominates early in infection, while X4 dominance appears later on, and increased thymic destruction has been associated with X4 viruses (reviewed in [85]).

### 4.2. Apoptosis and Senescence

Although the plasticity and capacity for regeneration of the immune system is prodigious, it may have boundaries. Thus, it becomes increasingly plausible that a cell can undergo a limited number of divisions and in the end will be trapped in growth arrest and immunological senescence, referred to as the Hayflick limit (reviewed in [86]). In the setting of HIV infection, this becomes relevant due to the increased production and turnover of cells. A possible way to determine the replicative history is to measure the length of the telomeres, which shorten by every cell division. The enzyme telomerase can compensate for this shortening, and indeed HIV-infected patients have been found to have shorter telomere length and dysregulated telomerase activity [87–89]. Short telomeres can lead to chromosome instability, involving growth arrest and apoptosis. Thus, not surprisingly HIV-infected patients present with elevated levels of apoptosis [90], and both early and late apoptotic CD4+ cells are more prevalent in patients with CD4+ cell counts <500 cells/*μ*L [90–94]. The relevance of markers of immune exhaustion and senescence in relation to immune reconstitution is confirmed by the findings of the expression of the activation associated T-cell molecule programmed death-1 (PD-1). PD-1 conveys inhibitory signals to T cells (reviewed in [95]), and PD-1 is selectively upregulated by exhausted T cells during chronic viral infection [96]. Elevated levels of PD-1 in INR compared to normal responders have been reported [80, 97]. Also, PD-1 expression has been shown to be negatively correlated to CD4+ cell count, and PD-1 expressing T cells are more prone to programmed cell death ligand-mediated inhibition of T cell proliferation [97].

Finally, chronic infection with cytomegalovirus (CMV) has been associated with immunological senescence, and a high proportion of T cells specific for CMV and CMV-viremia is associated with a low CD4+ cell count and increased mortality ([98], reviewed in [99]). 

### 4.3. Pro- and Anti-Inflammatory T Cells

During recent years, the understanding of immune responses has changed tremendously by the discovery of T-cell subsets with pro- and anti-inflammatory properties. Th17 cells are T cells with proinflammatory properties, while regulatory T cells (Tregs) are anti-inflammatory. Tregs play a crucial role in sustaining tolerance to self-antigens [100, 101] and suppressing T-cell activation resulting in downregulation of immune activation, including reduction in antitumor immunity, graft rejection, and graft-versus-host disease ([102], reviewed in [103]). Finally, the role of Tregs in chronic viral infections, including HIV, has gained massive interest due to their immunosuppressive capabilities. Tregs themselves are CD4+ cells and susceptible to HIV infection [104]. Therefore, the absolute number of Tregs declines with disease progression, while the frequency of Tregs tends to increase and remains high on HAART [105–108]. Thus, in a prospective study Tregs were measured in 26 HIV-infected patients before and after HAART and compared to healthy controls. The level of Tregs was found to be elevated in patients compared to controls, and this level did not change despite 6 months of HAART [106]. Tregs are believed to be able to downregulate chronic immune activation in HIV infection making Tregs a key element in the understanding of the interaction between the host immune system and HIV (reviewed in [109]). However, Tregs might be beneficial as downregulators of the unbeneficial immune activation or, in contrast, they might have a harmful effect downregulating HIV-specific responses. So far, it is not clear whether Tregs accelerate or delay HIV infection.

IL-17-producing Th17 cells are closely related to Tregs. Th17 cells and Tregs share a reciprocal maturation pathway and function together in opposing ways to control the inflammatory response to infection. While Tregs inhibit autoimmunity, Th17 cells play a role in the induction of autoimmune tissue injury [110]. During acute SIV infection the rapid depletion of Th17 cells and a disturbed balance of Th17 cells and Tregs are associated with subsequent high IA and disease progression [111]. Likewise, in HIV-infection the loss of balance between Th17 cells/Tregs may play a part in inducing microbial translocation and chronic immune activation [112] (reviewed in [113]). The importance of a well-regulated balance between Tregs and Th17 cells is demonstrated by a maintained balance between Tregs and Th17 cells in HIV controllers [114] (reviewed in [115]). Finally, recent data from our own lab show disturbances in the Treg- and Th17 cell compartments as well as in the balance between them in INR, suggesting an impact on immune reconstitution [116].

### 4.4. Secondary Lymphatic Tissue

CD4+ cell depletion occurs in the blood as well as in the secondary lymphatic tissue (SLT) of lymph nodes (LNs) and gut-associated lymphatic tissue (GALT) where the majority of the CD4+ cells reside. A vast number of cells are lost during primary infection, and by the time the infection has reached a chronic stage; more than 50% of the CD4+ cells in the LN are lost [117, 118]. With a possible damage of primary lymphatic tissue (LT) (i.e., thymic tissue and bone marrow) in mind, it seems reasonable to consider damages to SLT as a consequence of HIV infection as well, suggesting this early massive depletion as a determinant for the level of immune reconstitution following HAART. Thus, it has been proposed that HIV damages the structures in the lymphatic tissue that help sustain the normal CD4+ cell population, replacing the functional space with collagen. It was found that the greater the amount of the collagen-deposition, the lower the CD4+ cell count, and the smaller the number of naive CD4+ cells [119]. Furthermore, the amount of the collagen-deposition in LN has proven to be predictive for the degree of the immune reconstitution [120]. Also, LN biopsies from HIV and SIV-infected individuals show breakdown of the lymph node architecture and evidence of apoptosis [121].

These findings are consistent with HIV as a causative agent in damage to SLT. In light of this, it is worth noticing that SLT serves as viral reservoirs, including a pool of latently infected, resting CD4+ cells, which is believed to be a major impediment to the eradication of HIV [122]. While HAART rapidly reduces viral load in the blood, viral production is still detectable in SLT [123, 124], and it would be interesting to ascertain whether the pool of latently infected cells influences immune reconstitution. So far, it has been shown that the level of immune reconstitution is associated with certain types of cellular reservoirs. Thus, proviral DNA primarily persists in central memory cells in patients with a good immune reconstitution, while patients with a poorer reconstitution mainly host HIV proviral DNA in transitional memory cells [125]. This suggests that the viral reservoir influences immune reconstitution, and therefore it seems of interest to identify treatment strategies in addition to HAART with the ability to suppress the viral production in SLT, possibly leading to limited destruction of LT and a better immune reconstitution.

Another aspect is the fact that infection with HIV leads to redistribution of CD4+ cells between blood and lymphatic tissue. Thus, it has been demonstrated that HIV binds to resting CD4+ cells and upregulates L-selectin causing the cells to home from the blood into LN at enhanced rates [126, 127]. This has lead to the homing theory, which offers an explanation for the loss of CD4+ cells due to cells leaving the blood and entering the LT (reviewed in [128]). Thus, it would be interesting to evaluate the amount of CD4+ cells outside the blood in SLT in INR, which may reveal accumulation of CD4+ cells. Indeed accumulation of Tregs has been found in SLT compared to peripheral blood in untreated HIV-infected patients [129].

## 5. Clinical Implications: Relevance for Clinical Care

Treatment with HAART reduces the risk for development of AIDS and death. Consequently, a relative increase in morbidity and mortality has been described. Despite efficient treatment with HAART and suppression of viral replication, HIV-infected individuals have increased risk of morbidity and mortality when compared to uninfected population controls [130]. The reason for this is multifactorial. Thus, HIV itself is the leading cause (reviewed in [131]). However, HAART has been shown to cause metabolic and atherosclerotic changes (reviewed in [132, 133]). Furthermore, lifestyle-related factors such as increased alcohol consumption, smoking, drug-abuse, and poverty are all associated with being HIV infected (reviewed in [134–137]). Finally, among HIV-infected patients coinfection with hepatitis B and C is more common because hepatitis and HIV share transmission routes.

However, there are great differences in the risk of morbidity and mortality among different groups of HIV infected patients. Higher risk of opportunistic diseases and death was discovered to be associated with relative immunodeficiency in the SMART study [138], and INRs in particular have increased risk of opportunistic infections and long-term morbidity and mortality [11–13, 139]. Thus, INRs have a much higher risk of opportunistic diseases and death. In the UK CHIC study, the number of deaths/100 person years of follow-up was a hundred times higher in the group of patients with CD4+ cell counts below 50 cells/*μ*L compared to CD4+ cell counts above 500 cells/*μ*L [140]. Contrary, HIV-infected patients without risk factors and optimal response to HAART seem to have a mortality comparable to HIV negative individuals [141]. Importantly, rates of death increase substantially with declining CD4+ cell counts at baseline along with the extent of immunological deficiency prior to the period of sustained suppression of viral replication [11–13, 142]. For this reason, patients diagnosed with HIV in an advanced state of the disease (HIV late-presenters) are in higher risk of being INR (reviewed in [143]). Also, the lower the CD4 nadir, the shorter it takes for the CD4+ cell count to drop during treatment interruption [144].

Non-AIDS morbidity comprises of diseases such as cardiovascular disease (CVD), cancer, renal disease, hepatic disease, and osteoporosis. Thus, HIV-infected patients suffer more from clinical as well as subclinical atherosclerotic disease compared to the general population, and atherosclerotic (CVD) is a leading cause of death in HIV-infected patients [145]. HIV infection is associated with immune activation and inflammation. Inflammation in turn may lead to vascular damage and dysfunction increasing the risk of CVD (reviewed in [146]). Furthermore, HAART may increase the risk of myocardial infarction [147]. Either way, a consistent relation between low CD4+ cell counts and increased risk of CVD morbidity and mortality has been shown in a number of studies (reviewed in [148]). Thus, in a cross-sectional study of 1331 HIV-infected women and 600 HIV-infected men, the subclinical carotid artery lesions and common carotid artery intima-media thickness were measured by ultrasound. A low CD4+ cell count was found to be independently associated with an increased prevalence of carotid lesions [149]. The reason for this increased risk in INR is unknown, but the increased inflammation seen in patients with poor immune reconstitution as previously described seems like a more plausible reason than does the CD4+ cell count itself.

Likewise, HIV-infected patients have increased risk of cancer compared to the general population. It is well known that initially low and decreasing CD4+ cell counts during the year prior to cancer diagnosis are predictive of AIDS-defining malignancies (ADMs) such as Kaposi sarcoma and non-Hodgkin lymphoma as shown in the CASCADE study [150]. Also, the COHERE study showed that during HAART higher CD4+ cell counts are protective for the development of non-Hodgkin lymphoma [151]. Furthermore, non-AIDS-defining malignancies (non-ADM) such as anal cancer are more common in immune compromised patients (reviewed in [152]). In the D:A:D study the relationship between deaths due to ADM and non-ADM was determined, and immunodeficiency was evaluated. In a large observational cohort study including 23, 437 patients that were followed prospectively, a low CD4+ cell count was found to be predictive of death from both ADM and non-ADM in HIV-infected patients [153]. Likewise, a low current CD4+ cell count was shown to be associated with an increased incidence of certain non-ADM by the EuroSIDA group [154]. In conclusion, as expected ADMs are closely related to the CD4+ cell count. However, non-ADMs are related to the CD4+ cell count as well, presumably due to a poor immune function, especially since one study found that 48.3% of all non-ADMs were virus related [154]. All together HIV-infected patients have increased risk of developing malignancies, and more so in the group of INR.

Finally, the HIV-associated neurocognitive disorders (HANDs) have become an area of increasing interest as HIV infected patients become older and live longer. In the CHARTER study, a cross-sectional, observational study of 1,555 HIV-infected patients on HAART, the frequency and associated features of HAND were determined. Fifty-two percent of the total sample had neuropsychological impairment with higher rates in groups with greater comorbidity burden. The lowest impairment rate occurred in patients with a CD4 nadir and a current CD4+ cell count above 200 cells/*μ*L [155]. In general, a history of a low CD4 nadir seems be one of the strongest predictors of impairment [155, 156]. Future studies should identify whether early disease events (e.g., profound CD4 decline) may trigger chronic CNS changes, and whether early HAART prevents or reverses these changes.

In summary, there remains no doubt that a full immunological recovery is essential in order to reduce morbidity and mortality among HIV-infected patients. However, obtaining this goal requires improving earlier diagnosis and additional treatment to HAART. Several supplementary immune-based therapies to enhance immune reconstitution are under investigation using cytokines, hormones, and growth factors.

## 6. Therapeutical Possibilities

### 6.1. Optimizing HAART

**Figure 2:**
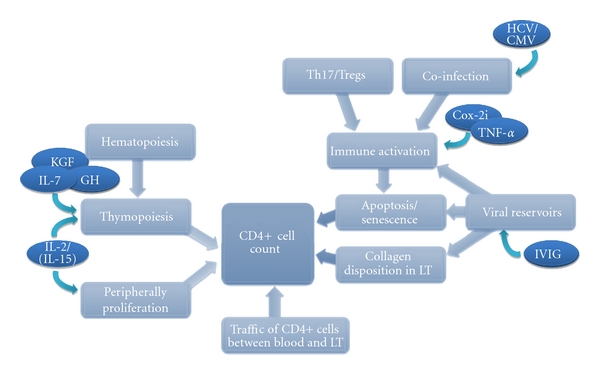
Therapeutical possibilities improving immune reconstitution. KGF: keratinocyte growth factor; IL: interleukin; GH: growth hormone; HCV/CMV: treatment of hepatitis C virus and cytomegalovirus; Cox2i: cyclooxygenase inhibitor; TNF: tumor necrosis factor; IVIG: intravenous immunoglobulin.

Optimizing HAART may be a possibility to increase CD4+ cell counts in HIV-infected INR (systematically reviewed in [157]), and recent data on new drug modalities such as CCR5-antagonists, integrase inhibitors, and fusion inhibitors increase the relevance of this matter. HIV uses CCR5 as a coreceptor for cell entry, which has led to development of several CCR5-antagonists to impede HIV infection. Currently, only Maraviroc is FDA-approved for treatment of HIV infection. The phase III randomized clinical MOTIVATION study demonstrated that addition of Maraviroc to HAART in pretreated patients resulted in a significant increase in CD4+ cell counts as well as reduced viral load [158]. Furthermore, administration of Maraviroc seems to result in decreased immune activation [159]. Underlining these findings, a meta-analysis of phase II/III clinical trials testing CCR5-antagonist in treatment-experienced HIV patients demonstrated significant increase in CD4+ cell counts by adding CCR5-antagonist [160], clearly suggesting a potential of drugs targeting CCR5 for optimizing immune reconstitution in INR. Raltegravir targets the HIV integrase, which facilitates the integration of the genetic material into the hosts DNA, and is at present the only FDA-approved integrase inhibitor. The potential of raltegravir has proven effective in the large phase III BENCHMRK study [161] showing increased CD4+ cell counts and reduced viral loads. However, two randomized clinical studies testing the effect of raltegravir in INR did not demonstrate an additional effect on immune reconstitution [162, 163]. Several clinical trials testing other integrase inhibitors are ongoing (http://www.clinicaltrials.org/), and a large phase III study comparing intensification of HAART with raltegravir and elvitegravir in treatment-experienced patients demonstrated no significant difference between the two drugs in regard with immune reconstitution [164]. Finally, enfuvirtide is the only FDA-approved fusion inhibitor. It impedes fusion of HIV to the target cell by binding to gp41. Two multicenter phase III studies (TORO 1/2) documented an effect of enfuvirtide in combination with HAART in treatment-experienced patients on both CD4+ cell count and viral load [165, 166]. However, a large randomized multicenter study (ANRS130) demonstrated no additional effect of supplementary enfuvirtide to HAART on CD4+ cell counts in treatment-naive late-presenters with low CD4+ cell count [167]. Thus, at present Maraviroc seems to be the most promising drug for HAART intensification. However, larger prospective studies assessing the effect in INR are needed to estimate the clinical benefits of HAART intensification (Figure 2).

### 6.2. Interleukin-2 (IL-2)

Several strategies to improve immune reconstitution in HIV patients have been considered during the last decade. Most of these strategies aim to increase thymus activity and/or peripheral proliferation. Of all suggested strategies supplementary treatment with recombinant human (rh)IL-2 is the best described. Thus, several studies have shown that combination therapy with rhIL-2 and HAART increases CD4+ cell counts in HIV patients compared to HAART alone (reviewed in [168]). The increased level of CD4+ cells is long-lasting and is caused by peripheral proliferation, although increased thymopoiesis may contribute as well [169]. Despite increased CD4+ cell counts, supplementary treatment with rhIL-2 did not result in clinical benefit for the treated patients, which was demonstrated in two large randomized prospective clinical trials (ESPRIT, 4111 patients, and SILCAAT, 1695 patients). The ESPRIT study revealed an increased risk of grade 4 clinical event in the IL-2-treated group. However, these data did not illustrate the (adverse) effects of supplementary rhIL-2 treatment in INR as median CD4+ cell counts were >400 cells/*μ*L in the ESPRIT study, and <200 cells/*μ*L in the SILCAAT study. In fact, stratified results for patients with a CD4+ cell count <200 cells/*μ*L in the SILCAAT study showed a nonsignificant decreased risk of adverse effect in the IL-2 treatment arm and still a significant increase in CD4+ cell count. A small study confirmed increased CD4+ cell count in INR after supplementary rhIL-2 treatment and demonstrated enhanced immune function ex vivo [170]. Also, a higher incidence of HIV-related clinical events has been observed among INR receiving HAART alone than among subjects receiving HAART plus IL-2 [171]. In conclusion, at present supplementary rhIL-2 has no place as a therapeutic agent in the treatment of HIV infection. However, it cannot be completely ruled out that INR may benefit, but it must be considered with great caution, and further studies are warranted to conclude whether any clinical benefit exists for supplementary IL-2 treatment in INR.

### 6.3. IL-7

As described, IL-7 is an essential regulator of the T-cell homeostasis. Administration of rhIL-7 to HIV-infected patients is being tested in several ongoing clinical trials (clincialtrials.gov, reviewed in [172]). Animal studies [173] and phase I/II studies report that IL-7 is well tolerated, but rhIL-7 administration causes a transient increase of viral replication [174, 175]. However, concomitant HAART counteracts this adverse effect and supplementary rhIL-7 treatment has proven safe [174, 175]. Subcutaneous administration of single dose of rhIL-7 (3–100 *μ*g/kg) [174] as well as intermittent doses (3 or 10 *μ*g/kg) [175] results in significant dose-dependent increase in CD4+ cell counts. This increase includes both central memory cells and naive cells. Importantly, administration of rhIL-7 has been shown to increase levels of RTE, numbers of TRECs, as well as increase naive CD4+ cell counts resulting in a broadening of immunological repertoire, thus indicating increased thymopoiesis [176–178]. Furthermore, preliminary data from the INSPIRE 2 study indicate that administration of rhIL-7 induces expansion of CD4+ cells in the gut mucosa due to increased expression of homing receptors [179] as well as lower expression of PD-1 suggestive of reduced immune activation. Thus, administration of rhIL-7 as supplementary treatment shows great promise. However, as lymphopenic HIV-infected patients present with physiological increased concentration of IL-7 [54, 55], exogenous IL-7 administration might be futile. Fortunately, animal studies [173] and phase I/II clinical trials reveal a significant increase in CD4+ cell count in individuals with low CD4+ cell counts and high IL-7 concentrations (reviewed in [172]). This finding may be due to the fact that circulating levels of IL-7 after rhIL-7 administration are much higher than physiological levels. Data from larger randomized clinical trials are warranted to determine the potential effect of adding IL-7 to HAART for immune reconstitution in INR, bearing the result of supplementary administration of IL-2 in mind.

### 6.4. IL-15

IL-15 is a cytokine that is structurally comparable to IL-2 and regulates proliferation and activation of T cells, and IL-15 has therefore been considered as a potential therapeutic agent in HIV infection. Treatment with IL-15 has primarily been investigated in murine and simian models [180, 181] with conflicting results. Combining IL-15 and HAART has proven to increase CD4+ cell and CD8+ cell counts in SIV-infected rhesus macaques (RMs) [182]. However, another study reported no effect of IL-15 administration on CD4+ cell counts but only on CD8+ cell counts and NK cells [181, 183]. Furthermore, administration of IL-15 in acute SIV infection resulted in increased viral load and increased disease progression [183], and similarly adding IL-15 to HAART in SIV-infected RMs resulted in decreased CD4+ cell counts. In conclusion, adding IL-15 to HAART is expected to result in limited benefit for INR.

### 6.5. Growth Hormone (GH)

Studies with supplementary therapy with GH in patients with HIV infection have originally been conducted to examine whether GH could be used a therapeutic agent for HIV-associated wasting and lipodystrophy (reviewed in [184, 185]). However, treatment with rhGH supplement to HAART has demonstrated to increase CD4+ cell counts compared to HAART alone in randomized, prospective clinical trials [186–190], and further clinical trials are ongoing (http://www.clinicaltrials.gov/). Furthermore, the supplementary rhGH given subcutaneous in doses from 0.7 to 3 mg/day resulted in an increase in thymic size in alignment with an increase in RTE, TREC number, and naive CD4+ cells [186–190], indicating an increased thymopoiesis. The patients included in these studies had median CD4+ cell counts <400 cells/*μ*L [187, 190] and 350 cells/*μ*L [186], respectively, and may therefore include a group of INR. However, adverse effects such as carpal tunnel syndrome, arthralgia, glucose intolerance, or cancer progression were frequent and result in a limited use of rhGH as a therapeutic agent. A potential strategy to reduce adverse effect is by using GH releasing factor (GHRF), which seems to cause fewer adverse effects (reviewed in [191, 192]). However, so far the effects of treatment with GHRF have primarily been addressed to lipodystrophia [192], (http://www.clinicaltrials.gov/), and indeed clinical trials testing the effect of GHRF on immune reconstitution are warranted.

### 6.6. Keratinocyte Growth Factor (KGF)

KGF causes proliferation and differentiation in thymic epithelial cells, and KGF pretreatment in mice and in rhesus macaques after myeloablative irradiation has proven to enhance thymopoiesis and increase thymic output [193–195]. However, a phase I/II randomized placebo-controlled study, originally designed to assess the effect of 40–60 *μ*g/kg KGF per day on graft versus host disease (GVHD) in 100 patients undergoing allogenic hematopoietic stem cell transplantation, showed no effect of KGF on absolute lymphocyte count [196, 197]. Thus, the addition of KGF to enhance immune reconstitution in INR on HAART is theoretically plausible, and results are awaited from a randomized phase II study testing the effect of palifermin (recombinant human KGF) injection at doses from 20 to 60 *μ*g/kg per day in HIV patients on HAART with CD4 count <250 cells/*μ*L (http://www.clinicaltrials.gov/).

### 6.7. Immune Suppression

The impact of immune activation is described previously. Several strategies have been suggested to reduce immune activation. New biological immunomodulators (inhibitors of TNF-alfa, IL-6, IL-1) for autoimmune diseases may prove to be therapeutic possibilities to suppression of immune activation in HIV infection. However, use of such immunomodulators must be done with great care taking into account the increased risks of opportunistic infections. Currently, there is limited experience, but HIV-infected patients with an autoimmune disease have been treated with TNF-*α* inhibitors with acceptable results for patients with CD4+ cell counts >200 cells/*μ*L (reviewed in [198, 199]). A small study described reduced immune activation and increased CD4+ cell count in treatment naïve patients after intravenous immunoglobulin (IVIG) (0.4 g/kg) [200]. However, this was not confirmed in another small study reporting no effect of high dosage IVIG (30 gram for five days) in treatment-experienced patients [201, 202], but interestingly IVIG administration resulted in a reduction of HIV reservoirs in CD4+ cell counts after treatment which may be a contributor to the failure of immune recovery in INR. Chronic coinfection with other infectious agents is a potential cause of increased immune activation in HIV-infected patients. Decreased immune activation has been documented after therapeutic clearance of HCV infection with interferon-*α* and ribavirin [203]. Likewise, a randomized clinical trial including 30 HIV-infected patients with CMV coinfection documented a decrease in chronic immune activation after CMV treatment in HIV patients with CD4+ cell counts <350 cells/*μ*L [204]. However, no difference in CD4+ cell count and HIV load was found.

### 6.8. Microbial Translocation

Microbial translocation has been suggested as a cause of immune activation and CD4+ cell depletion in HIV-infected patients [205–207]. High levels of 16S rDNA during therapy have been shown to be associated with reduced increases in the CD4+ cell counts [208], and heightened circulating lipopolysaccharide be associated with plasma enterobacterial DNA [209]. Thus, microbial translocation may be a potential target to decrease immune activation. Probiotics have been tested in randomized clinical trial in HIV-infected patient with CD4+ cell counts above 200 cells/*μ*L; however the results were disappointing [210]. Furthermore, the effects of hyperimmune bovine colostrums on CD4+ cell counts in INR were tested in a randomized clinical trials including 75 patients [163]. No change in immune activation or CD4+ cell counts was found. Thus, strategies to improve immune reconstitution in INR by modulation of microbial translocation are yet to emerge.

### 6.9. Cox-2 Inhibitor

Finally, another approach suggested to reduce immune activation is cyclooxygenase type 2 (Cox-2) inhibitors which have been tested in clinical trials with promising results. Treatment with Cox-2 inhibitors clearly decreases immune activation in HIV-infected patients [211–213], and combination treatment with HAART and Cox-2 inhibitor resulted in increased CD4+ cell counts [212, 213]. These studies were conducted in HIV-infected patients with median CD4+ cell counts >400 cells/*μ*L, and larger clinical trials assessing the effect of Cox-2 inhibitors in INR are needed to uncover a potential for optimizing immune recovery.

## 7. Conclusion and Future Directions

Following the introduction of HAART, the prognosis and life expectancy for HIV-infected patients has changed tremendously. Thus, patients with optimal immune reconstitution and lack of comorbidity have a life expectancy almost comparable to HIV negative individuals. However, rates of morbidity and mortality including both AIDS- and non-AIDS-related events increase substantially with persistent low CD4+ cell counts. Therefore, increased morbidity and mortality persist in patients who do not achieve full immune reconstitution, in particular in INR.

INRs have immunological dysfunctions in both production and destruction of CD4+ cells. A well-functioning bone marrow, a large thymus with adequate function, and a high output of naïve cells are all critical components for production of CD4+ cells and immune reconstitution. It is plausible that dysfunctions in one or more of these parameters contribute to the low CD4+ cell counts in INR. Also, INRs have higher levels of immune activation and apoptotic cells indicating a higher loss of CD4+ cells. With the known significant impact of immune activation on the prognosis in HIV-infected individuals, it is reasonable to conclude that a high level of immune activation is a contributing factor to poor immune reconstitution as well. Furthermore, a dysregulated balance between pro- and anti-inflammatory T cells in INR may have an influence on immune reconstitution, but definitive documentation is lacking. Finally, it is likely that disruptions in the secondary lymphatic tissue may contribute to lack of immune reconstitution. However, with the present level of knowledge, it is difficult to determine whether these immunological disturbances are reflecting a poor immunological reconstitution rather than causing them. Only well-designed large prospective studies can help clarify this.

So far, a range of supplementary treatment to HAART has been suggested to improve immune reconstitution. The only thoroughly investigated candidate has been IL-2 that unfortunately proved not to be beneficial for clinical outcome. Several candidates seem promising including supplementary treatment with IL-7, GH releasing analogues, and possibly Cox-2 inhibitors. Furthermore, using Maraviroc as an integrated component of HAART does seem to result in higher CD4+ cell counts, but at present, the possibilities of improving the immune reconstitution in INR using supplementary treatment are limited. Some predictive factors can be avoided. Early diagnosis could be improved, reducing the risk for a low CD4 nadir, and coinfection with hepatitis C can be treated. However, understanding and improving immune reconstitution in HIV-infected patients remains an important field of research.
